# Optimized formulation and processing protocol for a supplementary bean‐based composite flour

**DOI:** 10.1002/fsn3.244

**Published:** 2015-05-15

**Authors:** Catherine T. Ndagire, John H. Muyonga, Reddy Manju, Dorothy Nakimbugwe

**Affiliations:** ^1^ Department of Food Technology and NutritionMakerere UniversityKampalaUganda; ^2^ Department of Food Science and Human NutritionIowa State UniversityAmesIowa

**Keywords:** Antinutrients, common beans, digestibility, Response Surface Methodology, supplementary food

## Abstract

Protein‐energy malnutrition is the most serious nutritional body depletion disorder among infants and young children in developing countries, attributable to inadequate energy and nutrient intake, partly due to high dietary bulk of weaning and infant foods. The gruels fed to children are typically of low nutrient and energy density due to the low flour incorporation rate required for drinking viscosity. The aim of this study was to develop a nutritious product, based on common dry beans and other grains, suitable for supplementary feeding. The optimal processing conditions for desired nutritional and sensory attributes were determined using Response Surface Methodology. For bean processing, soaking for 6, 15, or 24 h, germination for 24 or 48 h, and cooking under pressure for either 10 or 20 min were the independent variables. The processed bean flour's total polyphenol, phytic acid and protein content, the sensory acceptability of the bean‐based composite porridge and its protein and starch digestibility were dependent variables. Based on product acceptability, antinutrients and protein content, as well as on protein and starch digestibility, the optimum processing conditions for the bean flour for infant and young child feeding were 24 h of soaking, 48 h of malting, and 19 min of steaming under pressure. These conditions resulted in a product with the highest desirability. The model equations developed can be used for predicting the quality of the bean flour and the bean‐based composite porridge. Bean optimally processed and incorporated with grain amaranth and rice flours of a ratio of 40: 30: 30, respectively, resulted into flour with high energy, mineral, and nutrient density of the final porridge. The composite is well adaptable to preparation at rural community level. The use of these locally available grains and feasible processes could make a great contribution to nutrition security in sub‐Saharan Africa and other developing countries.

## Introduction

In Uganda, infant and childhood malnutrition due to inadequate energy and nutrient density has been associated with the high viscosity of gruels fed to children (Kikafunda et al. 2006). Therefore, there is need to develop less viscous nutrient and energy‐dense foods to supplement infants' and young children's diets. Through blending of common staples and application of suitable processing procedures, it is possible to develop highly acceptable products with enhanced energy and nutrient density, from foods commonly grown in developing countries.

Beans are high in nutrients yet rarely utilized for porridges to feed young children. On the other hand, the utilization of nutrients from beans is limited by antinutrients. Most of the porridges served to young children especially in rural Uganda are made from sole flours yet blending common staples enhances energy and nutrient density. The aim of this study was to determine the optimum formulation and processing conditions for a bean‐based composite flour with reduced levels of total polyphenol and phytate, high protein and starch digestibility, and a high level of consumer acceptability.

As the common dry bean is highly nutritious, the digestibility of its macronutrients and bioavailability of its micronutrients is limited by antinutritional factors such as trypsin inhibitors, lectins, polyphenols, and phytic acid (Gibson et al. 2006), whose removal requires appropriate processing (Uebersax 2006; Esenwah and Ikenebomeh 2008) such as soaking (Adeparusi 2001), germination, and cooking (Zia‐ur and Salariya 2005). Improving sensory acceptability of supplementary foods is important because undesirable sensory properties of foods are key dietary factors affecting energy and nutrient intake of children (Arimond and Ruel 2004). Cooking generally improves palatability of foods (Ramakrishna et al. 2006). Blending (WHO, 2004) and processing, including germination (Herlache 2007) and starch pregelatinization (Scattergood and Cunningham 1999) have been used to produce flours that make high nutrient and energy‐dense gruels for infant and young child diet supplementation.

The aim of the study was also to determine the optimum formulation and optimize processing conditions for the bean flour. Specific objectives were to reduce antinutrients (total polyphenol and phytates), improve protein and starch digestibility of porridge from the developed composite flour, evaluate the pasting properties of the composite flour and the acceptability of its porridge, and compare the energy and nutrient (protein and mineral) density of porridge from the bean‐based composite flour to those of widely consumed maize and millet porridges.

## Methods

### Preprocessing of flours

To process bean flour, common dry bean (var. K131) was obtained from National Crops Resources Research Institute (NaCCRI) in Uganda, cleaned by manual sorting, batch washed three times with potable water and soaked for 6, 15, and 24 h in water (200% volume for volume). The soaked beans were drained and spread on a plastic tray lined with a thick wet cloth then covered with another wet piece of cloth and left to germinate. The germinated beans were rinsed and steamed under pressure (15 psi at 121°C for 10 or 20 min) using a domestic pressure cooker. The steamed beans were then spread on a metallic tray and dried in a fan oven (GALLENKAMP, Hotbox Oven with Fan, SG93/08/850, UK) at 75°C for 6 h to a final moisture content of 6%. The dried beans were then milled using a Wondermill grinder (Grain of Truth Bread Company, Arlington, VA 22922). To process rice flour, white rice (super brand) was procured from a retail shop in Kampala city, cleaned by manual sorting, and milled using the above mill into fine flour. To process grain amaranth flour, golden colored grain amaranth (*Amaranthus spp*.) was procured from farmers in Kamuli district, cleaned by manual sorting and pan‐roasted at 250°C for 5 min, to produce a distinct roasted flavor (Bahika 2008). The roasted amaranth grains were also milled into fine flour using the above mill.

### Determining the optimum incorporation level of beans, grain amaranth, and rice into the composite flour

Concept 4 creative software (CREATIVE FORMULATION CONCEPTS, LLC, Annapolis, MD 21401) was used to determine the optimum level of incorporation of beans, grain amaranth, and rice flours into the composite to significantly contribute to the protein and energy requirements of children aged 2–5 years (1046 kcal/day and 902 kcal/day for male and female children aged 2 and 3 years and 1742 kcal/day and 1642 kcal/day for male and female children aged 4 and 5 years and, 13 g/day for both male and female children aged 2 and 3 years and 19 g/day for those aged 4 and 5 years) (Whitney and Rolfes 2002). The common dry bean with 23.6% protein complimented rice and grain amaranth of 6.5 and 13.6% protein, respectively (USDA, 2011).

### Optimization of the bean flour processing protocol

The desirability function approach (DFA) was used to simultaneously optimize the composite porridge's acceptability, protein and starch digestibility, and the bean flour's total polyphenol, phytate, and protein content. These are very important characteristics of meals fed to infants and young children. The desirability function approach method incorporates desires and priorities for each of the variables and the maximum desirability is 1 (Mepha et al. 2007).

### Optimization research design

Each independent variable was varied as shown in Table 1. A D‐Optimal design (Table 2) was used to determine the influence of three independent variables (soaking time [*X*_1_], germination time [*X*_2_], and steaming time [*X*_3_]) on the nutritional quality and consumer acceptability of bean flour and bean/rice/amaranths composite porridge.

**Table 1:** Processing variables and their levels in the D‐Optimal design

Variables	Symbol	Coded variables		
		−1	0	1
Soaking time (h)	*X*_1_	6	15	24
Germination time (h)	*X*_2_	0	24	48
Steaming time (min)	*X*_3_	0	10	20

**Table 2:** D‐Optimal design used to optimize levels of soaking, germination, and cooking of beans

Run	Coded values			Actual values		
	Soaking time (h)	Germination time (h)	Steaming time (min)	Soaking time (h)	Germination time (h)	Steaming time (min)
1	−1	−1	−1	6	0	0
2	−1	−1	−1	6	0	0
3	−1	−1	1	6	0	20
4	−1	1	−1	6	48	0
5	−1	1	1	6	48	20
6	1	−1	−1	24	0	0
7	1	−1	1	24	0	20
8	1	1	−1	24	48	0
9	1	1	1	24	48	20
10	1	1	1	24	48	20
11	1	0	0	24	24	10
12	−1	0	0	6	24	10
13	0	−1	0	15	0	10
14	0	1	0	15	48	10
15	0	0	−1	15	24	0
16	0	0	1	15	24	20
17	0	0	0	15	24	10
18	0	0	0	15	24	10
19				0	0	0

The variables were expressed individually as a function of the independent variables. The data were fitted to the following second‐order approximating model (eq. (1)):

(1) Y=B0+∑i−1kBiXi+∑i−1kBiiXi2+∑i−1i<jkBijXiXj+ε

Where Y is the response function, *ε* is the random error, *B*_o_ the center point of the system, B_i_, B_ii_, and B_ij_ represent the coefficients of the linear, quadratic, and interactive effects, respectively, and X_i_, $X_{i}^{2}$ and X_i_X_j_ represent the linear, quadratic, and interactive effects of the independent variables (soaking time, germination time, and steaming time), respectively.

### Optimizing acceptability of composite porridges

Porridges were prepared by dissolving 150 g of the bean‐based composite flour in 400 mL of cold water, adding the resulting paste to 450 mL of boiling water, and boiling for 8 min with constant stirring. Sensory attributes (color, flavor, thickness, appearance, smell, taste, and texture) of the bean‐based composite flour porridges, from each run (Table 2) were evaluated using a 9‐point hedonic scale (1 =  dislike extremely and 9 =  like extremely). The sensory panel comprised of six Ugandan students at Iowa State University that were familiar with porridge characteristics and had consented to be part of the study. Protocols for conducting sensory evaluation in this study were approved by the Institutional Review Board at Iowa State University.

### Protein digestibility

In vitro protein digestibility was determined using pepsin‐pancreatin enzyme system (Saunders et al. 1973; Chavan et al. 2001). Protein content of the sample was determined with a NITROGEN (N)‐ANALYZER (Elementar Americas, Inc., Mt. Laurel, NJ) before and after digestion and digestibility was calculated using the formula:

%protein digestibility=(A−B)/(A)

where *A* is % protein in the sample before digestion and *B* is % protein in sample after enzyme digestion.

### Starch digestibility

Starch digestibility was determined using the Megazyme resistant starch assay K‐RSTAR 05/2008 (Megazyme, 2008).

### Total polyphenol and phytic acid content determination

Polyphenol content was measured colorimetrically as Gallic acid equivalents using the Folin‐Ciocalteau colorimetric method (Singleton and Rossi 1965; Memnune et al. 2009). Phenolic compounds were extracted from samples using methanol/water/acetic acid solution (70:30:5), Folin‐Ciocalteau reagent was added and Catechin and Gallic acid equivalents were determined as absorbance at 765 nm.

Phytic acid content was determined using the anion‐exchange method as total phosphorus (AOAC, 1999).

### Sensory Evaluation of porridges made from bean‐based composite, millet, and maize flours

Sensory evaluation of the porridge attributes (overall acceptability, appearance, smell, taste, texture, color, aftertaste, flavor, and after taste) was done at the School of Food Technology, Nutrition and Bio‐engineering sensory laboratory. The panel consisted of 75 males and females aged 18–50 years, familiar with porridge characteristics. The porridges were rated on a 9‐point Hedonic scale where 9 is like extremely and 1 is dislike extremely. The panelists who had consented to participate in the study also gave additional comments on the porridges.

### Pasting properties of bean‐based composite, millet, and maize flours

Pasting properties of bean‐based composite (finely milled using a WONDERMILL GRINDER, Grain of Truth Bread Company, Arlington, VA 22922), finely milled millet, and maize flours (procured from a retail shop in Kampala city) flours were determined using Rapid Visco Analyzer (RVA‐4, Newport Scientific, Warrie‐wood, Australia). Peak viscosity, breakdown, final viscosity, set back, peak time, and pasting temperature were read from the pasting profile with the aid of thermocline for windows software (Newport Scientific, 1998).

### Proximate analysis

The moisture, crude protein, ash, fat, mineral, total carbohydrate, total fat, and gross energy contents of the bean‐based composite flour were determined using standard methods. Moisture content was determined by oven drying overnight at 98°C (AOAC (Association of Official Analytical Chemists) 1999); crude protein content with a NITROGEN (N) ‐ANALYZER (Elementar Americas, Inc., Mt. Laurel, NJ) (USDA, 2009); ash content by igniting a dried, ground sample in a furnace at 600°C for 2 h to oxidize all organic matter (AOAC (Association of Official Analytical Chemists) 1999); gross energy using the bomb calorimetry method. Fat content was determined using the Soxhlet method (AOAC (Association of Official Analytical Chemists) 1999); total carbohydrate using Colorimetric Quantification of Carbohydrates assay (Wiley 2001); and mineral profile by Hach Method (HACH, 1997) using the HACH MODEL 21400 DIGESDAHL APPARATUS, Colorado.

### Nutrient density determination

Porridges of the bean‐based composite, millet, and maize flours were prepared at different concentrations in water and boiled for 10 min. The porridges were then placed in a water bath maintained between 54 and 56°C, the recommended consumption temperature for porridges by young children (Dawn et al. 1994). The viscosity (centipoises, cP) of the porridges was measured using a Brookfield Viscometer (Model DVII Rheometer V2.0 RV; Middleboro, Massachusetts), with spindle number 63 at a shear rate of 1.5 rpm. These parameters gave the highest torque values compared to other combinations (Table 3). Flour rates that resulted in porridge viscosities of 2500–3000 cP, which are suitable for infant and young child consumption (Mosha and Svanberg 1983) were determined. Energy and nutrient density (protein and minerals: phosphorus, potassium, magnesium, calcium, sodium, iron, copper, and zinc) of the flours were calculated for flour rates resulting porridge viscosities of 2500–3000 cP.

**Table 3:** Viscosity (cP) and torque (%) of 10% flour rate of the bean‐based composite porridge at 55°C using spindle size 63

RPM	Mean viscosity	Mean percentage torque
0.3	4222.5 ± 96.0	41.9 ± 0.6
0.6	2228 ± 31.2	55.48 ± 0.6
1.5	1437.5 ± 10.8	80.9 ± 2.6
3	EEEE	EEEE

The bean‐based composite flour attained this viscosity at 15%, millet flour at 8% while maize flour at 7% flour (w/v). ± their standard deviation, EEE‐error.

### Data analysis

To optimize the bean flour processing protocol, data were analyzed by Response Surface Methodology (RSM) procedures using Design‐expert statistical software (DX 6.0; Stat‐Ease, Inc., MN; 2003). Statistical parameters used to relate input variables to responses were *P*‐value and *R* ^2^ of the models. Data for sensory attributes (appearance, smell, taste, and consistency/mouth feel) and pasting properties (peak, breakdown, final, setback viscosities) of the bean‐based composite, millet, and maize porridges and flours, respectively, were compared using the Statistical Package for Social Scientists 16.0 software program (SPSS software, release 16.0, SPSS Inc.). Means, standard deviations were determined, analysis of variance was performed to calculate significant differences in treatment means and the TUKEY technique (*P* < 0.05) was used for separation of means using SPSS.

## Results

Different combinations of common dry bean, grain amaranth, and rice flour exhibited varying protein and energy contents (Table 4). The formulation with 40, 30, and 30% bean, grain amaranth, and rice flours was most adequate since it exhibited relatively balanced protein and energy contents.

**Table 4:** Formulations with grain amaranth, bean, and rice flours and their energy and protein contents as predicted by Concept 4 Creative Formulation Software

Rice	Amaranth	Beans	Energy/100 g	Protein (%)
80	10	10	362	9.2
70	20	10	363	10.1
60	20	20	359	11.6
50	20	30	360	12.3
10	80	10	366.5	13.9
20	60	20	362	14.2
10	10	80	329.9	20.9
30	10	60	346	17.6
**30**	**30**	**40**	**353**	**15.6**
30	20	50	350	15.92

The formulation in bold font was chosen as optimal and used for this study because its predicted protein and energy content were relatively balanced.

**Figure 1:**
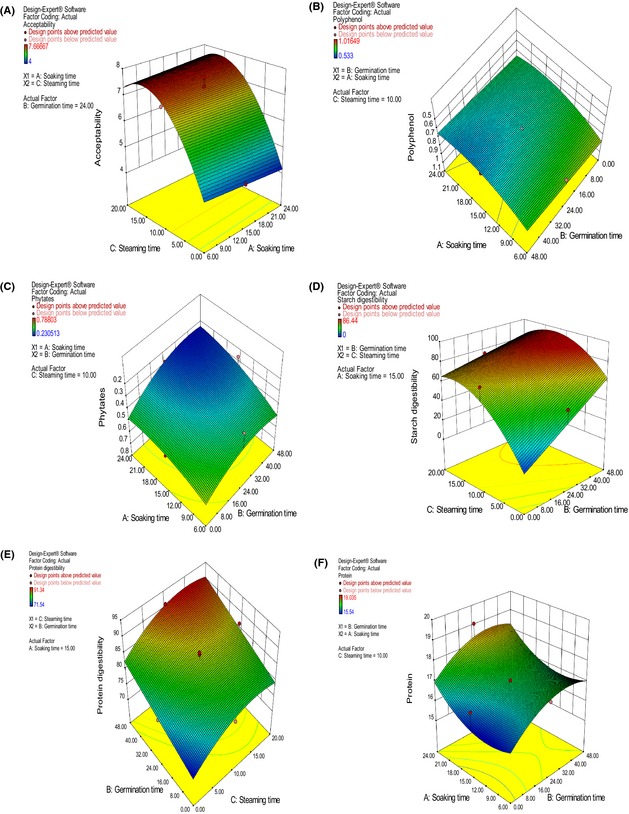
Response surface plots showing effect of soaking, germination, and steaming on bean‐based composite porridge's overall acceptability (A), bean flour's total polyphenol content (B), bean flour's phytates content (C), bean‐based composite porridge's starch digestibility (D), bean‐based composite porridge's protein digestibility (E), and bean flour's protein content (F).

The responses of the preprocessed bean flour and the bean‐based composite porridge varied between different combinations of soaking, germination and steaming time (Fig. 1, Table 5).

**Table 5:** D‐ Optimal design arrangement and responses

Run	Variables			Responses					
	Sk	Gn	St	Tp (%)	Pa (%)	Pt (%)	OA (av.s)	Std (%)	Ptd (%)
1	6	0	0	0.94	0.61	15.54	4.83	1.47	71.54
2	6	0	0	0.93	0.63	15.69	4.83	1.16	72.01
3	6	0	20	0.86	0.58	15.61	7.33	68.96	78.53
4	6	48	0	0.84	0.57	16.80	4.33	61.29	81.71
5	6	48	20	0.72	0.48	17.90	7.33	84.54	90.96
6	24	0	0	0.64	0.48	17.39	4.00	10.06	71.88
7	24	0	20	0.76	0.45	16.10	7.50	55.77	79.02
8	24	48	0	0.83	0.24	17.92	4.17	64.91	82.07
9	24	48	20	0.53^a^	0.23^a^	18.18	7.67^a^	84.44	91.34^a^
10	24	48	20	0.56	0.23^a^	15.56	7.50	86.32^a^	89.78
11	24	24	10	0.76	0.29	19.04^a^	7.17	77.36	84.33
12	6	24	10	0.78	0.34	17.00	7.17	73.30	84.12
13	15	0	10	0.78	0.51	16.48	7.17	70.00	77.97
14	15	48	10	0.63	0.27	16.45	7.17	84.91	90.78
15	15	24	0	0.56	0.35	16.73	4.33	48.22	76.44
16	15	24	20	0.66	0.33	16.95	7.33	75.15	86.47
17	15	24	10	0.68	0.38	16.76	7.62	73.87	86.40
18	15	24	10	0.67	0.38	17.05	7.33	74.05	87.01
19	0	0	0	1.02	0.79	16.62	4.33	0	71.61

Sk, soaking time (h); Gn, germination time (h); St, steaming time (min); Tp, total polyphenol content; Pa, phytic acid; OA, overall acceptability; Std, starch digestibility; Ptd, protein digestibility.

a Desired response value for required product attributes.

### Predictive models

Equation (2) defines the effect of processing on porridge's overall acceptability.

(2) $$\begin{array}{c} \text{Acceptability}\;=\;7.28\;-\;0.033X_{1}\;+\;1.55X_{3}\;+\;0.164X_{1}X_{3}\;-\\ \;1.40X_{3}^{2}\;\left(R^{2}\;=\;0.99\right) \end{array}$$

Soaking and steaming time had a negative and positive linear effect, respectively, on acceptability. Soaking and steaming time had positive interactive effects on acceptability while steaming time had a significant negative quadratic effect on acceptability. Steaming time significantly (*P* < 0.0001) increased the acceptability of the composite porridge. Acceptability of the porridges made from cooked beans was low and did not significantly change with soaking time with a mean score of 4.40 on a 9‐point hedonic scale.

Soaking, germination, and steaming time affected total polyphenol content of the bean flour (eq. (3)).

(3) $$\begin{array}{c} \text{Total polyphenol}\;=\;0.67\;-\;0.062X_{1}\;-\;0.047X_{2}\;-\;0.033X_{3}\;-\\ \;0.052X_{2}X_{3}\;+\;0.091X_{1}^{2}\;\left(R^{2}=0.80\right) \end{array}$$

Soaking, germination, and steaming time had a negative linear effect on the bean flour's total polyphenol content. Steaming and germination time had a significant negative interactive effect while only soaking had a positive significant quadratic effect on the total polyphenol content of the bean flour. Increased soaking and germination time significantly reduced polyphenol content of the bean flour and the model was significant (*P* = 0.0003).

Equation (4) defines the significant predictive model for bean flour's phytates content (*P* < 0.0001)

(4) $$\begin{array}{c} \text{Phytates}\;=\;0.33\;-\;0.098X_{1}\;-\;0.090X_{2}\;-\;0.031X_{1}X_{2}\;+\\ \;0.048X_{1}^{2}\;+\;0.08X_{2}^{2}.\;\left(R^{2}=0.92\right) \end{array}$$

Soaking and germination time were the only significant model terms, showing negative linear and interactive effects and positive quadratic effect on phytate content of the bean flour.

Starch digestibility of the bean‐based composite porridge was affected by germination and steaming time of the bean flour (eq. (5))

(5) $$\begin{array}{c} \text{Starch digestibility}\;=\;75.62\;+\;18.96X_{2}\;+\;19.87X_{3}\;-\\ \;9.68X_{2}X_{3}\;-\;20.83X_{3}^{2}\;\left(R^{2}=0.96\right) \end{array}$$

Steaming and germination time of the beans had positive linear effects while germination and steaming time had negative interactive effect on starch digestibility of the bean‐based composite flour porridge. Only steaming time had a quadratic effect on starch digestibility of the bean‐based composite flour porridge which was negative.

The relationship between soaking, germination, and steaming time of beans and the bean‐based composite porridge's protein digestibility is represented by equation (6) and resulted in a significant quadratic model (*P* < 0.0001)

(6) $$\begin{array}{c} \text{Protein digestibility}\;=\;85.46\;+\;0.056X_{1}\;+\;5.59X_{2}\;+4.07X_{3}\;-\\ \;4.2\times 10^{-4}X_{1}X_{2}\;+\;0.032X_{1}X_{3}\;+\\ \;0.51X_{2}X_{3}\;-\;0.38X_{1}^{2}\;-0.71X_{2}^{2}\;-\\ \;3.63X_{3}^{2}\;\left(R^{2}=0.98\right) \end{array}$$

All the three variables (soaking, germination and steaming time of beans) had positive linear effects, only soaking and germination time had negative interactive and quadratic effects on the bean‐based composite porridge's protein digestibility. Positive interactive effects existed between soaking and steaming time and between germination and steaming time. Steaming time also had a negative quadratic effect on protein digestibility.

In equation (7) the predictive model was significant (*P* = 0.0001) and showed both linear and quadratic effects of soaking and germination time of beans on its flour's protein content.

(7) $$\begin{array}{c} \text{Protein content}\;=\;17+0.62X_{1}\;+\;0.65X_{2}\;+\;0.76X_{1}^{2}\;-\;0.75X_{2}^{2}\\ \;(R^{2}=0.79) \end{array}$$

Both soaking and germination time had positive linear effects on protein content while soaking time had a positive quadratic effect and germination had a negative quadratic effect.

### Optimal processing conditions

The processing conditions chosen as optimum were those that resulted in a product with the highest desirability (0.94) (Table 6). These were: soaking beans for 24 h, germinating for 48 h, and steaming under pressure for 19 min. These processing conditions resulted in bean flour with low phytate content (0.22%), low total polyphenol content (0.57%), moderate protein content (18.27%) and bean‐based composite flour with high starch digestibility (87.73%), high protein digestibility (91.6%), and high overall acceptability (7.67 on a scale of 1–9).

**Table 6:** Optimal solutions

Number	Soaking time (hr)	Germination time (hr)	Steaming time (min)	Starch digestibility (%)	Protein %	Phytates (%)	Polyphenol (%)	Acceptability (mean scores)	Desirability
1	24.00	48.00	18.68	87.73	18.27	0.22	0.57	7.67	0.9423
2	24.00	47.04	18.56	87.60	18.30	0.22	0.58	7.67	0.9410
3	24.00	42.70	17.77	87.39	18.42	0.22	0.61	7.72	0.9327
4	24.00	38.77	13.47	89.60	18.49	0.23	0.65	7.67	0.9136

Proximate compositions (moisture, protein, ash, fat, gross energy, nonresistant starch, resistant starch, total starch, total carbohydrates, and minerals (phosphorus, potassium, magnesium, calcium, sodium, iron, copper, and zinc)) of the bean‐based composite flour were determined and it was found to have substantially good quantities of energy and nutrients (Table 7).

**Table 7:** Nutritional value of the optimal bean‐based composite flour

Nutrient	% composition
Moisture	5.4
Protein	13.2
Ash	10.3
Fat	10.4
Nonresistant starch	43.7
Resistant starch	8.2
Total starch	52
Total carbohydrates	64.4

Energy	
Gross energy	435.8 kcal/100 g (1823.4 kJ/100 g)

Minerals	mg/100 g
Phosphorus	317
Potassium	623
Magnesium	157
Calcium	170
Sodium	6.8
Iron	12.8
Copper	2.2
Zinc	6.3

The mean scores of porridge attributes (color, overall appearance, smell, taste, flavor, mouth feel, thickness, after taste, and overall acceptability) on the 9‐point Hedonic scales were compared (Table 8). Overall appearance scores of the porridges were highest for bean‐based composite and lowest for millet. There was no significant difference (*P* > 0.05) between bean‐based composite and maize porridges, which in turn were significantly preferred to millet porridges. Scores for flavor were not significantly different (*P* > 0.05) between the bean‐based composite porridge and maize porridge yet it varied significantly between millet and bean‐based composite porridge, with the bean‐based porridge being superior. Overall acceptability scores for the different porridges were not different (*P* > 0.05) between the bean‐based composite and maize porridges. The millet porridge's overall acceptability was significantly different from and inferior to that of both millet and bean‐based composite porridges. Overall acceptability for the bean‐based composite porridge was superior to those of both maize and millet porridges. Mean acceptance scores for color, smell, taste, texture, after taste and mouth feel did not differ significantly (*P* > 0.05) between bean‐based composite and maize porridge yet were significantly different (*P* < 0.05) and were superior to those of millet porridge.

**Table 8:** Mean scores and their standard deviations of sensory attributes of the bean‐based composite, maize, and millet porridges on a 9‐point hedonic scale

Attribute	Mean score ± their Standard Deviations^a^		
	Bean‐based composite porridge	Maize porridge	Millet porridge
Overall appearance	7.2 ± 0.8^ac^	7.0 ± 0.9^a^	5.9 ± 1.5^ab^
Color	7.3 ± 0.8^a^	7.3 ± 1.0^a^	6.0 ± 1.5^b^
Smell	6.7 ± 0.9^a^	6.5 ± 0.9^a^	5.8 ± 1.4^a^
Texture	7.2 ± 0.8^a^	7.5 ± 0.8^a^	5.5 ± 1.5^b^
Taste	7.3 ± 1.0^a^	7.3 ± 0.9^a^	5.8 ± 0.9^b^
Flavor	6.7 ± 0.9^ac^	6.3 ± 1.2^a^	5.8 ± 1.0^ab^
Mouth feel	7.1 ± 0.8^a^	7.1 ± 1.0^a^	5.7 ± 1.5^b^
After taste	6.9 ± 0.7^a^	6.6 ± 1.1^a^	5.3 ± 1.6^b^
Overall acceptability	7.4 ± 0.7^a^	7.3 ± 0.7^a^	6.2 ± 0.9^b^

Figures in a row with the same letter as their first superscript are not significantly different. Figures in a row with the same letter as their second superscript are not significantly different (*P* < 0.05). Rows were compared to establish how different sensory attributes were between the three porridges.

a Scores 1 – Dislike extremely, 2 – Dislike very much, 3 – Dislike moderately, 4 – Dislike slightly, 5 – Neither like nor dislike, 6 – Like slightly, 7 – Like moderately, 8 – Like very much, 9 – Like extremely.

**Figure 2:**
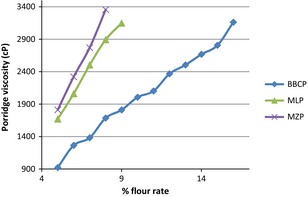
Variation in porridge viscosities (cP) of the bean‐based composite, millet, and maize porridges at varied flour rates

Use of spindle size 63 of the Brookfield viscometer at 1.5 rpm resulted in higher torque values and was therefore used in the study. The bean‐based composite flour attained viscosity in the range of 2500–3000 at 15%, millet flour at 8% while maize flour at 7% flour (w/v), (Fig. 2).

**Figure 3:**
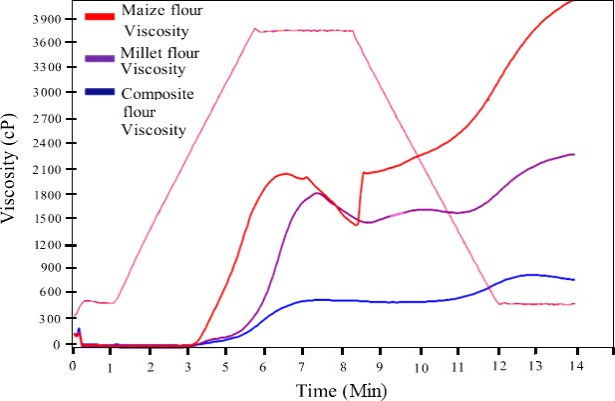
Rapid Visco Analyzer profiles of bean‐based composite, millet, and maize flours.

On comparison of the bean‐based composite, maize and millet flours' RVA parameters: peak viscosity, breakdown viscosity, final viscosity, setback viscosity, peak time, and pasting temperature, the bean‐based composite had the lowest peak, breakdown, final and set back viscosity (profile COMPOSITE 1 viscosity in Fig. 3, Table 9). These were significantly lower than those of millet and maize flour. As the maize flour's peak viscosity was higher but not significantly different from that of millet flour (*P* > 0.05), its break down, final, and set back viscosities were higher and significantly different from those of millet flour (P < 0.05). The results showed that there were significant differences (*P* < 0.05) in the pasting temperature of the different flours. The bean‐based composite flour exhibited a significantly higher pasting temperature than both millet and maize flour. The bean‐based composite flour also exhibited the highest peak time that was not significantly different from that of millet (*P* > 0.05). Maize porridge exhibited a significantly lower peak time (*P* < 0.05) than both millet and the bean‐based composite flours. The mean final viscosity values were lowest for the bean‐composite and highest for millet flours.

**Table 9:** Means and standard deviations of pasting properties of the bean‐based composite, maize, and millet flours

Pasting property	Bean‐based composite flour	Maize flour	Millet flour
Peak viscosity (cP)	493 ± 19.3^a^	1986.7 ± 79.7^bd^	1798.7 ± 19.8 ^cd^
Breakdown viscosity (cP)	24.3 ± 3.8^a^	553.3 ± 75.3^b^	336 ± 49.5^c^
Final viscosity (cP)	740.3 ± 17.9^a^	4096.7 ± 138.0^b^	2231.3 ± 38.2^c^
Set back viscosity (cP)	271.7 ± 2.5^a^	2663.3 ± 121.6^b^	768.8 ± 43.6^c^
Peak time (min)	6.4 ± 0.1^a^	5.49 ± 0.04^b^	6.3 ± 0.0^a^
Pasting temperature (°C)	92.2 ± 0.1^a^	76.22 ± 0.5^b^	89.3 ± 0.5^c^

Figures in a row with the same letter as their first superscript are not significantly different. Figures in a row with the same letter as their second superscript are not significantly different (*P* < 0.05). Rows were compared to establish how different pasting properties were between the three flours.

Energy, protein, and mineral (phosphorus, potassium, magnesium, calcium, iron, copper, zinc, manganese) densities (as served) of the bean‐based composite porridge were superior to densities of maize and millet porridges except for sodium that was more in maize porridge than millet and the bean‐based composite porridges (Table 10). Energy density of the bean‐based composite porridge (65.8/275.2 kcal/kJ/100 mL) was higher than that for millet and maize porridges (29.8/124.9 and 25.6/106.9 kcal/kJ/100 mL, respectively). Likewise, the protein density of the bean‐based composite porridge (1.98 g/100 mL) was higher than that for millet and maize porridges (0.9 and 0.7 g/100 mL, respectively).

**Table 10:** Nutrient density of 100 mL of different porridges

Nutrient	Maize porridge	Millet porridge	Bean‐based composite porridge
Energy (kcal/kJ)	25.6/106.9	29.8	65.8
Protein (g)	0.7	0.9	2.0
Minerals (mg)			
Phosphorus	14.7	22.8	47.6
Potassium	20.1	17.9	93.5
Magnesium	8.9	9.5	23.6
Calcium	0.5	1.1	25.5
Sodium	2.5	0.3	1.0
Iron	0.2	0.3	1.9
Copper	0.02	0.04	0.3
Zinc	0.2	0.2	1.0

Values calculated for the bean‐based porridge are based on chemical analysis while those for maize and millet porridge are based on nutrient database values of USDA (2011).

## Discussion

Sensory characteristics of a food are key factors in determination of energy and nutrient intake of infants and young children as they consume less of foods with inferior sensory characteristics than those of superior sensory characteristics. Desirable sensory attributes are key factors for foods as it determines acceptability and consumption. In this study, acceptability of the porridge at different levels of bean flour processing increased with increasing steaming time. Cooking improves palatability of foods (Ramakrishna and Ramakrishna 2006). Improved palatability of food with cooking could be partly explained by reduction in phenolic compounds, known to be responsible for the bitterness and astringency of many foods and beverages (Delcour et al. 1984), by cooking (Habiba 2002). The observed improvement in acceptability with steaming time could be attributed to reduction in the levels of polyphenols, which are associated with undesirable beany flavors (Nwosu 2010). Roasting grain amaranth produced a pleasant flavor (Bahika 2008) playing a big role as a flavor improver of the porridge.

The optimized processing protocol reduced the levels of antinutrients in the product. Increased soaking and germination time led to reduced polyphenol content of the bean flour. Ramakrishna and Ramakrishna (2006) reported that malting was a more effective method for reducing polyphenols than the various cooking treatments. Decrease in phenolic compounds because of soaking and germination may be due to decomposition, covalent linkage to structural polymers, or entrapment within the solid endosperm matrix (Waniska 2000). Reduction in polyphenol content can also be attributed to leaching into soaking water (Afify et al. 2011). Phytate content of bean flour decreased with increased soaking and germination time from 0.79% to 0.23%. The observation agrees with the findings of (Akpapunam et al. 1996) who reported a 76 and 59% loss of phytic acid contents of soybean and bambara groundnut flours, respectively, upon malting for 120 h. The activity of phytase, an enzyme found in malt like barley and known to break down phytate increases 7.9‐fold during malting (Herlache 2007). The observed lowered phytate content of soaked and germinated beans can therefore be attributed to both leaching loss (Afify et al. 2011) and degradation due to increased phytase activity.

Starch digestibility of the bean‐based composite flour porridge increased with increased steaming and germination time. This may be attributed to action of amylases during germination and increase in ease with which starch gets digested after cooking since it breaks down fibrous cellulose. Alpha‐ and beta‐amylase enzymes present in malt break down carbohydrates to maltriose, maltose, and glucose (Herlache 2007). The phytate molecule, containing six phosphate groups, is highly charged, making it an excellent chelator and it can form insoluble complexes with proteins leading to reduced digestibility (Wedad et al. 2008). Magdi (2007) reported that malting significantly enhanced protein digestibility of Dolichos Lablab Bean. This was attributed to phytic acid hydrolysis as well as degradation of protein. Herlache (2007) also reported an increase in the activity of proteases during germination of cereals. Increase in protein digestibility can also be attributed to breakdown of long protein chains by proteases. Protein content of the bean flour apparently increased with increased germination time. Malting has been shown to yield apparent increases in grain protein content (Griffith and Castell‐Perez 1998)**.** The apparent increase in protein content resulting from germination has been linked to shift in dry matter through depletion of carbohydrates during germination (Wedad et al. 2008).

Starch in foods is responsible for their pasting properties thus viscosity. The high flour rate for bean‐based composite porridge is attributable its low starch content (51.97%), (Table 6). Since millet has lower starch content (69.88%) (Saunders et al. 1973) than maize (89.3%) (Mora‐Escobedo et al. 2004), its porridge gave a high flour rate than of maize porridge. The bean‐based composite flour porridge also had a low viscosity attributable to its low starch content. Since millet has lower starch content than maize, its flour gave a lower viscosity than of maize porridge. It is expected that soy‐based porridge would have a much higher flour rate compared to that of the bean‐based porridge since soy has much lower starch content (Birmingham et al. 1978).

Energy and nutrient density of infants and young children's feeds is a vital factor in their nutritional status as it determines energy and nutrient intake. The bean‐based composite porridge's higher nutrient density as compared to maize and millet (Table 9) is attributable to nutrient complementation as well as the higher flour rate used. The higher flour rate was possible because of the lower starch content. Millet porridge's nutrient density was higher than that of maize and this can be attributed to higher flour rate used for millet porridge than for maize porridge, because of the lower starch content of millet flour. The bean‐based porridge's higher nutrient density is attributable to its higher flour rate that is attributable to germination of the beans used for the composite flour during the preprocessing of the bean flour. It can also be attributed to compositing the bean flour with rice and grain amaranth flours where different ingredients complimented one another making an energy and nutrient‐dense formulation. Millet nutrient density was superior to that of maize porridge and this can be attributed to higher flour rate exhibited by millet porridge than maize porridge.

The relatively high viscosity exhibited by maize and millet starch is indicative that the flours are less suitable for infant and young child feeding as infant and young children require less viscous porridges. On the other hand, the low viscosity exhibited by the bean‐based composite starch is indicative that the flour is suitable for infant and young child feeding that requires less viscous porridges. The relatively low energy and nutrient densities exhibited by maize and millet porridges signifies that the porridges may not be suitable for infant and young child feeding that requires high energy and nutrient density. The high energy and nutrient density exhibited by the bean‐based composite porridge shows that the flour is suitable for infant and young child feeding that requires high energy and nutrient density.

## Conclusions

Soaking, germination, and steaming of bean flour result in significant improvements in the sensory and nutritional quality of the flour and increase its suitability for feeding infants and young children. The optimal processing conditions for dry beans are soaking for 24 h followed by germination for 48 h and steaming under pressure for 19 min, as they result in high acceptability and protein and starch digestibility as well as reduction in antinutrients. Surface Response Methodology (SRM) is a suitable approach to optimizing formulation for complementary foods and the model equations developed closely predicted the quality of the bean flour and bean‐based composite porridge incorporating the processed flour. Dry beans, when optimally processed and composited with other common grains result in a nutrient‐dense complementary food with the potential to contribute to reduction in macro and micronutrient malnutrition among infants and young children.

## Conflict of Interest

Catherine T. Ndagire, John H. Muyonga, Reddy Manju, and Dorothy Nakimbugwe do not have any conflicting interests.
